# Rate of opioid use disorder in adults who received prescription opioid pain therapy—A secondary data analysis

**DOI:** 10.1371/journal.pone.0236268

**Published:** 2020-07-23

**Authors:** Johannes M. Just, Norbert Scherbaum, Michael Specka, Marie-Therese Puth, Klaus Weckbecker

**Affiliations:** 1 Institute of General Practice and Interprofessional Care, Faculty of Health / Department of Medicine, University Witten/Herdecke, Witten, Germany; 2 LVR-Hospital Essen, Department of Addictive Behaviour and Addiction Medicine, Medical Faculty, University of Duisburg-Essen, Essen, Germany; 3 Department of Medical Biometry, Informatics and Epidemiology (IMBIE), University Hospital Bonn, Bonn, Germany; St. Michael's Hospital, CANADA

## Abstract

**Background and aims:**

Data on rates of prescription opioid use disorder (pOUD) in European countries is limited. The aim of this investigation was to analyze a representative population sample regarding the 1-year prevalence of opioid use disorder in patients who received prescription opioid pain therapy and to identify related risk factors.

**Design:**

Cross-sectional secondary data analysis

**Setting:**

Secondary data analysis based on data from the 2015 Epidemiological Survey of Substance Abuse (ESA 2015) in Germany

**Participants:**

German-speaking individuals living in private households aged 18 to 64 years were investigated. A total of 9204 individuals participated in the survey, resulting in a response rate of 52.2%.

**Primary and secondary outcome measures:**

Primary outcome measure was the weighted prevalence of pOUD in the subgroup of study participants who had received prescription opioids. Secondary outcome measure was an analysis of risk factors connected with pOUD in the same subgroup.

**Findings:**

A total of n = 9204 participants were included in the study of which n = 275 had received an opioid prescription in the last 12 months of which n = 54 were diagnosed with pOUD. The weighted 1-year prevalence of pOUD was 21.2% (mild: 14.7% | moderate: 3.5% | severe: 2.9%). Participants who had received opioid pain therapy had significantly higher odds of pOUD if they reported signs of depression (OR: 2.69; CI 95%: 1.13–6.38), inexplicable physical complaints (OR: 2.68; CI 95%: 1.14–6.31) or a psychiatric diagnosis (OR: 4.12; CI 95%: 1.36–12.43), and significantly lower odds of pOUD if they reported the use of non-opioid painkillers (OR: 0.27; CI 95%: 0.09–0.81).

**Conclusions:**

pOUD is a common phenomenon in working-age patients who receive prescription opioid pain therapy in Germany and may be related to the co-existence of psychosomatic and psychiatric disorders such as depression.

## 1. Introduction

The United States opioid epidemic which was initially driven by prescription opioids has developed into a crisis that now envelops heroin as well as illicitly manufactured fentanyl resulting in many deaths and extensive collateral harm to families as well as communities throughout the US [1]. As of now, there are no signs of a similar development in Europe and Germany although prescription opioid consumption is increasing despite a lack of documented effectiveness in the treatment of chronic non-cancer pain (CNCP) [2, 3].

Data from the European Monitoring Centre for Drugs and Drug Addiction show stable numbers for problematic opioid use and overdose deaths throughout the EU between 2000 and 2018 [3]. Against that stands a notable increase in prescription opioid consumption in Europe and especially Germany. In 2016, the German prescription opioid market was the second largest of all OECD countries, with defined daily doses per one million inhabitants only being surpassed by the USA by roughly one third [4].

The current consensus of experts on addiction and pain therapy in Germany is, that there are no signs of an opioid epidemic on a national level in Europe and Germany, but the level of evidence that supports this consensus is low [5–9]. Still, there is little data with a focus on diagnosing addiction or misuse in patients receiving prescription opioids. Two recent, cross-sectional trials in Germany showed that a significant proportion of ambulatory patients with CNCP and long-term opioid therapy (LTOT) were at high risk of opioid misuse (31.5%) or had been diagnosed with prescription opioid use disorder (pOUD) (26.5%), yet both trials lacked in size and generalizability [10, 11]. Additional analysis from larger datasets would be useful to assess the situation in Germany.

A suitable dataset for this purpose is the Epidemiological Survey of Substance Abuse 2015 (ESA 2015) [12]. It represents a valuable resource to further our knowledge on rates of pOUD in patients receiving prescription opioids in Germany. It contains all relevant diagnostic criteria for pOUD and was representative for German-speaking individuals living in private households aged 18 to 64 years.

The aim of this study was to perform a cross-sectional data analysis using data from the ESA 2015. Primary outcome measure was the weighted prevalence of pOUD in the subgroup of study participants who had received prescription opioids. Secondary outcome measure was an analysis of risk factors connected with pOUD in the same subgroup.

## 2. Methods

### 2.1 Sample and procedure

The ESA is regularly performed by “infas” (Institut für angewandte Sozialwissenschaften GmbH) on behalf of the German Federal Ministry of Health. Aim of the ESA 2015 was to perform a systematic survey of the use of psychoactive substances in the general population in Germany thereby building a basis for the improvement of prevention strategies as well as an assessment of the impact of legal and regulatory interventions on consumption patterns [12].

The sample of the ESA 2015 consisted of 9204 German-speaking participants aged 18 to 64 years who lived in private households. The sample was drawn between March and July 2015 from an estimated age group population of approximately 52 Million, using a random two-stage selection procedure. At the first stage, 254 sample points (cities, communities) were drawn. Then, the target population was randomly chosen from the sample points’ population registers. Paper-and-pencil, telephone and internet-based methods were used to collect the data (response rate 52.2%). Using design as well as post-stratification redressement weights, the sample was adjusted to the distribution of the general population according to federal state, size of the community, sex, year of birth, and school education. Overall, 5090 women (49.6%) and 4114 men (50.4%) with an average age of 38.3 years (SD = 14.7) participated in the ESA 2015 [12].

Participants were not involved in the development of the study design or outcome measures. Participation was voluntary and could be terminated by participants at any time. Data was collected anonymously and kept confidential.

The ESA was approved by the ethics committee of the German Psychological Society (DGPs; Reg.-No: GBLK06102008DGPS).

We included all participants who reported that they had taken prescription opioids within the last 12 months and had received them via a regular prescription. The primary question used in the survey was: “Have you taken opioid painkillers in the last 12 months? (yes/no)”. It wasn’t documented how frequently or for what period of time prescription opioids were taken. Further questions focused on the source from which prescription opioids were obtained. Participants who obtained prescription opioids from illicit sources only were excluded. Patients who had taken opioid painkillers were the asked for diagnostic criteria of pOUD.

Data holders gave the authors permission to use the data for analysis, but did not further participate in the conduction and publication of this study.

### 2.2 Choice of diagnostic concept/primary outcome variable

We chose the concept of opioid use disorder (OUD) as proposed in the Diagnostic and Statistical Manual of Mental Disorders, Fifth Edition (DSM-5) as diagnostic criterion [13].

OUD is defined as a problematic pattern of opioid use leading to clinically significant impairment or distress, as manifested by at least two diagnostic criteria, occurring within a 12-month period.” OUD consists of eleven diagnostic criteria, although, in patients taking prescription opioids under appropriate medical supervision, only nine diagnostic criteria are applicable as recommended in DSM-5 (see appendix for all diagnostic criteria). OUD is divided into three levels of severity: mild OUD (2–3 positive criteria), moderate OUD (4–5 positive criteria) and severe OUD (>6 positive criteria). In this study, we preferred the acronym pOUD = prescription opioid use disorder with the intention to make clear that we were studying patients under medical opioid therapy and not those who used prescription or non-prescription opioids from illicit sources only. In participants who stated that they had received additional opioids from another source (n = 17) all 11 diagnostic criteria were applied as we concluded that appropriate medical supervision was unlikely [13]. As OUD is a new diagnostic criterion, data on its reliability and validity is still needed [14].

Although the diagnosis OUD is not widely used in Germany, it is level of care in the US and therefore allows for comparison with international studies. Its advantages to other diagnostic criteria include a dynamic spectrum (mild, moderate and severe disorder) and likely a reduced social stigma compared to the term ‘addiction’ [13–15].

### 2.3 Secondary outcome-/ independent variables

Independent variables that were tested for association with the presence of pOUD were either used in their original bi- / multivariate or metric form or were transformed into a bivariate composite score. The ESA 2015, for instance, included the question if and how often participants had taken cocaine, amphetamine, marihuana and other illicit drugs within the last 12 months. Because of the limited size of our sample we developed a binary composite score “Illicit drugs taken within the last 12 months yes/no” in order to pool the information. Every patient who had at least once consumed an illegal drug within the last 12 months was labeled as “yes” in this new variable.

The variables used for testing were: age (metric), gender (male/female), International Standard Classification of Education (ISCED: low/middle/high), Net-household income (OECD modified equivalence scale) below poverty threshold (yes/no), living with partner (yes/no), currently employed (yes/no), additional prescription opioids from illicit source (yes/no), illicit drugs taken within the last 12 months (yes/no), more than one day spent being completely drunk within the last 12 months (yes/no), sleeping pill or tranquilizer taken within the last 12 months (yes/no), antidepressant taken within the last 12 months (yes/no), non-opioid painkiller taken within the last 12 months (yes/no), inexplicable physical complaints within the last 12 months (yes/no), depressed mood for at least two weeks within the last 12 months (yes/no), diagnosis of or treatment for psychiatric disease within the last 12 months (yes/no), traumatic event within the last 12 months (yes/no). All items were self-reported (e.g. “Within the last 12 months, have you been feeling sad or depressed almost daily for longer than two weeks?”). Variable selection was based on the available literature on likely risk factors for opioid addiction [16–19].

### 2.4 Data analysis

Analysis was performed using “IBM SPSS Statistics 26” following a predefined study protocol. Design and post-stratification weights were applied to the sample. The 1-year prevalence of pOUD among participants who had received prescription opioids, was estimated using descriptive statistics. We estimated ORs and 95% confidence intervals (CI) using a logistic regression to identify risk factors associated with pOUD compared to those without pOUD among participants who had received prescription opioids.

## 3. Results

A total of n = 9204 participants were included in the study of which n = 275 had received an opioid prescription in the last 12 months of which n = 54 were diagnosed with pOUD. The weighted 1-year prevalence of pOUD was 21.2% (mild: 14.7% | moderate: 3.5% | severe: 2.9%).

### 3.1 Demographic characteristics

Patients who had received prescription opioid pain therapy were generally older and showed a higher proportion of female participants than the complete study population (mean age 44.53 vs. 42.35 years; female gender 62.4 vs. 50%). Table 1 shows more detailed characteristics of participants who reported the use of prescription opioids at least once within the last 12 months.

**Table 1:** Demographic characteristics of participants who reported the use of opioids with a medical prescription within the last 12 months.

Characteristics of participants who reported the use of opioids with a medical prescription within the last 12 months, weighted % (n, unweighted)	
Fraction of total study population	3.6% (275)
Age	Mean: 44.53
	Range: 18–64
	SE: 0.81
Gender:	Female: 62.4% (182)
	Male: 37.6% (93)
Living without partner:	30.6% (100)
ISCED (International Standard Classification of Education):	Primary Education: 12.3% (32)
	Lower secondary Education: 69.6% (172)
	Higher secondary Education: 18.0% (71)
Currently unemployed:	26.1% (73)
Net-household income below poverty threshold	37.3% (77)

Data source: ESA 2015, Germany.

### 3.2 Main results

#### 3.2.1 Prevalence of pOUD

Out of the total study population (n = 9204), 3.5% (n = 275) of participants reported the legal use of prescription opioids which they had received from a doctor within the last 12 months. Of those, 21.2% (n = 54) met diagnostic criteria for pOUD (moderate and severe pOUD: 6.4%). Converted to the total population of the study, this corresponded to a 1-year prevalence of pOUD in Germany of 0.8% (moderate and severe pOUD: 0.2%). In the group of participants with pOUD, 7 out of 54 (15.3%) reported the additional use of illegal opioids like heroin within the last 12 months while only 3 out of 221 (3.8%) did so in the group of participants without pOUD. All reported percentages were weighted. Absolute and relative numbers including positive patients per criterion are given in Table 2.

**Table 2:** Weighted % of prescription opioid recipients according to OUD severity.

	No pOUD:		Mild pOUD:		Moderate pOUD:		Severe pOUD:			
Weighted % of population who received prescription opioids (n, unweighted)	78.8% (221)		14.7% (37)		3.5% (11)		2.9% (6)			
Number of positive criteria	0	1	2	3	4	5	6	7	8	9
Weighted % of Patient distribution (n, unweighted)	55.4% (159)	23.4% (62)	9.3% (23)	5.4% (14)	1.0% (4)	2.4% (7)	1.9% (4)	1.1% (2)	0% (0)	0% (0)

Data source: ESA– 2015, Germany.

The most frequently positive criteria were “Recurrent opioid use resulting in a failure to fulfill major role obligations at work, school, or home”, followed by “Recurrent opioid use in situations in which it is physically hazardous”. A complete list is displayed in S1 Table.

#### 3.2.2 Results of multiple regression analysis

We used a multiple regression model to identify risk factors associated with participants who reported the use of prescription opioids and met diagnostic criteria for pOUD compared to those who reported the use of prescription opioids but did not meet diagnostic criteria for pOUD. The model was sound, showing a Nagelkerke’s Pseudo-R^2^ of 0.38. All tested variables are shown in Table 3.

**Table 3:** Risk factors for opioid use disorder (weighted).

	Weighted percentage of OUD negative participants (n, unweighted)	Weighted percentage of OUD positive participants (n, unweighted)	Weighted OR (95% CI), adjusted
total	78.8% (221)	21.2% (54)	N/A
Gender: male	39.5% (75)	30.3% (18)	0.42 (0.15–1.21)
Age (mean, SE)	45,02 (0.92)	42,67 (1,72)	0.99 (0.96–1.02)
Living without partner	29.7% (78)	34.2% (22)	1.38 (0.54–3.53)
ISCED	N/A	N/A	N/A
Primary education	11.5% (22)	15.3% (10)	2.09 (0.46–9.44)
Lower secondary Education	69.2% (140)	71.4% (32)	1.27 (0.47–3.43)
Household income below poverty threshold	34.3% (57)	48.2% (20)	1.39 (0.54–3.61)
unemployed	26.5% (61)	24.6% (12)	0.52 (0.17–1.54)
Regular smoker	37.7% (59)	51.0% (21)	1.60 (0.64–4.00)
Received additional prescription opioids from other source	5.6% (13)	3.7% (4)	0.23 (0.03–1.89)
Illicit drugs taken within the last 12 months	13.2% (23)	20.4% (13)	0.64 (0.14–2.86)
has been very drunk at least once within the last 12 months	26.0% (60)	27.0% (21)	2.56 (0.60–10.92)
Sleeping pill or tranquilizer taken within the last 12 months	12.1% (25)	21.8%(12)	0.53 (0.19–1.52)
Antidepressant taken within the last 12 months	13.8% (23)	39.2% (19)	2.50 (0.77–8.16)
Non-opioid painkiller taken within the last 12 months	91.1% (200)	77.0% (44)	0.27 (0.09–0.81)
Inexplicable physical complaints within the last 12 months	32.8% (65)	62.9% (33)	2.68 (1.14–6.31)
Depressed mood for at least two weeks within the last 12 months	33.8% (62)	66.9% (34)	2.69 (1.13–6.38)
Diagnosis of or treatment for psychiatric or psychosomatic disease within the last 12 months	18.9% (41)	57.7% (27)	4.12 (1.36–12.43)
traumatic experience within the last 12 months	47.7% (100)	55.3% (28)	0.50 (0.18–1.39)

Logistic regression analysis, n = 275, df = 18; N = absolute number of participants; (%) = percentage within variable DSM-5—Score; OR = Odds ratio; (95% CI) = 95% Confidence Interval for Odds ratio, Sig. = Significance of OR. Data source: ESA– 2015, Germany.

## 4. Discussion

The weighted 1-year prevalence of pOUD was 21.2% (mild: 14.7% | moderate: 3.5% | severe: 2.9%). Participants who had received opioid pain therapy had significantly higher odds of pOUD if they reported signs of depression (OR: 2.69; CI 95%: 1.13–6.38), inexplicable physical complaints (OR: 2.68; CI 95%: 1.14–6.31) or a psychiatric diagnosis (OR: 4.12; CI 95%: 1.36–12.43), and significantly lower odds of pOUD if they reported the use of non-opioid painkillers (OR: 0.27; CI 95%: 0.09–0.81).

### 4.1 Potentials and limitations of the study

This study is the first to report the prevalence of pOUD in a representative, non-clinical population sample in Germany. Data from the ESA 2015 was generally well suited for the needs of our analysis. The data collection was methodically sound, its focus already aimed at addictive behaviours and the sample showed satisfactory generalizability regarding socio-demographic criteria after application of redressement weights [12]. Many addiction-related variables to characterize participants were collected during primary data collection and were therefore available for inclusion in the statistical model we used, covering several known risk factors for pOUD. Relevant risk factors that were not included in the survey concerned clinical data on opioid pain therapy like indication (e.g. cancer vs. non-cancer), duration and dosing. [16,19]

Limitations in generalizability include the age limitation of the group (18–64 years) and the inclusion of community-dwelling participants living in a household only, thereby excluding institutionalized persons like prisoners. However, this limitation may not be as relevant in Germany compared to other countries, as the incarceration rate is relatively low (Germany: 77 prisoners vs. USA: 655 prisoners per 100.000 citizens) [20]. In terms of validity, several limitations apply. Participants may have underreported addictive behavior due to social desirability bias. Recall bias may have led to an underreporting of opioid consumption in those who didn’t experience any distress in relation to opioid therapy, thereby increasing rates of pOUD in the population. Also, we chose a conservative approach in dealing with missing data und could therefore have underreported the prevalence of pOUD. Comparing participants with and without pOUD within the subgroup of prescription opioid users led to a relatively small sample size. In combination with the considerable number of covariates in the model, this led to wide CI. This leads to uncertainty regarding the strength and direction of several effects, especially where the CI crossed 1. Furthermore, questions have been raised regarding the general validity of the new DSM-5 definition for OUD itself, as diagnostic thresholds have been lowered compared to the DSM-4 [14, 15, 21]. Intentions and implications of the lowered threshold in relation to our results will be discussed in the following chapter.

### 4.2 Interpretation of main results

As a main result, a proportion of pOUD of 21.2% in patients who received prescription opioid pain therapy appears rather high, given the fact that the analysis is based on a survey of a representative population sample in a country in which there are no reports of an increase in opioid related mortality. This result may be closely related to the intent of the lowered threshold and labeling in the DSM-5. In comparison to former concepts (e.g. ICD-10 “Dependence”), OUD includes more patients as “affected”. Accordingly, Degenhardt et al. found good agreement between ICD-10 and DSM-IV dependence diagnoses and a moderate to severe OUD, while agreement was only fair to moderate when compared with any degree of OUD [21]. This may lead to the interpretation that, while 21.2% of opioid users showed signs of distress regarding their medication only 6.4% (moderate to severe pOUD) are likely to display a co-diagnosis with opioid dependence/misuse. Some overlap (15.3%) between the presence of pOUD and the use of illegal opioids was shown in our sample, but based on the method, no causal connection can be proven. Therefore, it could be argued that in those 15.3%, the label pOUD may be incorrect, as the illegal opioid may have been the driving factor in the participants OUD.

Extrapolating the proportion of pOUD into absolute numbers for the German population arrives at an estimated 416.000 individuals (0.8%) with pOUD of which appx. 104.000 (0.2%) show moderate to severe pOUD. As to why such a big number of patients with pOUD exists without increases in opioid-related mortality is perplexing. In a best case scenario, an opioid epidemic in Germany is prevented by protective environmental factors including compulsory health insurance with a well-financed and accessible primary care sector including specialized pain therapy, opioid maintenance therapy and specialized addiction therapy. Additionally, adequate regulatory restrictions for opioids exist, including a ban on direct to consumer marketing. In a worst case scenario, there is a black spot in diagnostics related to opioid induced deaths which may be linked to social and professional desirability, the low quality and underfunding of cause of death-examination in Germany and a monitoring focus on heroin use [8, 22]

In terms of procedure, we believe that all patients diagnosed with pOUD should receive increased attention by their attending physician as they have shown significant impairment or distress in relation to opioids. In addition, those with moderate to severe disorder may need specialized follow-up and therapy by an addiction specialist as the coincidence of an ICD-10 or DSM- IV dependence diagnosis is likely [21].

### 4.3 Findings on pOUD prevalence from other studies

The prevalence of at least one opioid prescription within the last 12 months (3.5%) is lower than that reported in other studies (4.1%) and is most likely related to the lower age and therefore lower prevalence of chronic pain in ESA 2015 participants [19]

Several studies from non-European countries have assessed the proportion of pOUD in populations with chronic pain which were similar to our results. They ranged between 21%, 23% and 41.3% in the USA as well as 20.8% in Australia and 26.5% in Germany [10, 16, 23, 24]. Unfortunately, the ESA 2015 did not assess the presence of chronic pain nor the indication for, or duration of opioid pain therapy. Therefore we could not evaluate the relevance of chronic pain as a contributing factor in our sample.

### 4.4 Risk factors for pOUD

Most of our results were in line with data from the USA [16–18]. Strong predictors for opioid misuse by chronic pain patients in the US included a personal history of illicit drug and alcohol abuse [16, 17]. Both factors showed a tendency towards an increased likelihood of pOUD in our study but results weren’t significant. Frequently discussed risk factors like poverty and a low educational status showed a tendency towards an increased likelihood of pOUD in our study, but results weren’t statistically significant either [18]. We did however find statistically significant correlations between mental health issues and an increased OR for pOUD, as has been discussed in US studies [16, 18]

The odds ratio for pOUD was increased significantly in participants who reported a psychiatric diagnosis, signs of depression and inexplicable symptoms. We interpreted all three factors as different aspects of depressive disorders. Depressive and somatoform disorders as well as chronic pain are often causally connected and/or comorbid conditions, each negatively affecting the other. Frequently, a diagnosis of depression is delayed by initial presentation with symptoms of somatization like dizziness or pain [25]. Additionally, patients on LTOT with comorbid depression are more likely to receive opioids [26]. This is a particular problem, as opioids may in some cases reduce symptoms of depression, but also put patients with depressive disorders at a high risk of pOUD [16,18]. Opioids were even used as a primary treatment for depression in the 19th century with good initial results but an often fatal long-term outcome [27]. Our findings may therefore point at a diagnosis and treatment gap: The underdiagnosis of primary or secondary depression in patients reporting with somatization symptoms like pain, and its unintentional mistreatment with opioids, resulting in pOUD.

We interpreted the significantly lower odds of pOUD in participants who reported the additional use of non-opioid painkillers as a sign of guideline-oriented pain therapy. Guideline-orientated opioid pain therapy includes the use of non-opioids and non-pharmacological interventions in combination with opioid pain therapy in the management of chronic pain. It may therefore decrease the need for high-dose LTOT which in turn is associated with a higher risk for pOUD [19, 28].

## Conclusion

Our results showed a 1-year prevalence of pOUD of 21.2% in patients who received prescription opioid therapy, despite low and stable rates of opioid overdose deaths in Germany. Our results suggest that the preconditions for opioid pain therapy may differ greatly between countries, a circumstance that stresses the importance of adapted national guidelines on opioid prescription.

We did find rather strong connections between psychiatric disorders, especially depressive disorders and pOUD. Therefore, additional emphasis should be put on depression screening in patients with CNCP and non-opioid and non-medical interventions should be prioritized.

Additional studies focusing on larger population samples, including more patient criteria like daily opioid dose, are desirable in order to get a more detailed picture of pOUD in Germany and Europe. Future interventions should focus on how to reduce proportions of pOUD in chronic pain without impairing pain therapy itself. Better diagnosis and therapy of comorbid psychiatric and psychosomatic disorders could be a key to achieving this goal.

## Supporting information

S1 Table Criteria for opioid use disorder and proportion of positive patients per question. (DOCX)
