# Correction: Rate of opioid use disorder in adults who received prescription opioid pain therapy—A secondary data analysis

**DOI:** 10.1371/journal.pone.0246146

**Published:** 2021-01-22

**Authors:** Johannes M. Just, Norbert Scherbaum, Michael Specka, Marie-Therese Puth, Klaus Weckbecker

In the Sample and procedure subsection of the Methods, there is an error in the first sentence of the first paragraph. The correct sentence is: The ESA is regularly conducted by the Institute for Therapy Research (IFT Institut für Therapieforschung).

Information is missing from the Acknowledgments. The correct Acknowledgments are as follows: We thank the IFT Institut für Therapieforschung for providing the data for this analysis.

There are errors in the Funding statement. The correct Funding statement is as follows: The 2015 Epidemiological Survey of Substance Abuse (ESA) was supported by funding from the German Federal Ministry of Health (project no. IIA5 –2514DSM200).
