# Supplementary material for: Rate of opioid use disorder in adults who received prescription opioid pain therapy—A secondary data analysis
Source: PLoS One. 2020 Jul 23;15(7):e0236268. doi: 10.1371/journal.pone.0236268 (PMC7377413; doi:10.1371/journal.pone.0236268)
Supplement: S1 Table — (DOCX) [file pone.0236268.s001.docx]

**S1 Table: Criteria for opioid use disorder and proportion of positive patients per question**

| Opioid use disorder: “A problematic pattern of opioid use leading to clinically significant impairment or distress, as manifested by at least two of the following criteria, occurring within a 12-month period.” | | Proportion of positives in % (absolute) |
| --- | --- | --- |
| 1 | Opioids are often taken in larger amounts or over a longer period of time than intended. | 9.4% (29) |
| 2 | There is a persistent desire or unsuccessful efforts to cut down or control opioid use. | 15.0% (35) |
| 3 | A great deal of time is spent in activities necessary to obtain the opioid, use the opioid, or recover from its effects. | 5.2% (14) |
| 4 | Craving, or a strong desire to use opioids. | 5.7% (14) |
| 5 | Recurrent opioid use resulting in a failure to fulfill major role obligations at work, school, or home. | 20.8% (51) |
| 6 | Continued opioid use despite having persistent or recurrent social or interpersonal problems caused or exacerbated by the effects of opioids. | 6.3% (15) |
| 7 | Important social, occupational or recreational activities are given up or reduced because of opioid use. | 11.5% (29) |
| 8 | Recurrent opioid use in situations in which it is physically hazardous | 14.9% (41) |
| 9 | Continued use despite knowledge of having a persistent or recurrent physical or psychological problem that is likely to have been caused or exacerbated by opioids. | 3.6% (6) |
| 10* | Tolerance, as defined by either of the following:  ● A need for markedly increased amounts of opioids to achieve intoxication or desired effect.  ● A markedly diminished effect with continued use of the same amount of an opioid. (Note: This criterion is not considered to be met for those taking opioids solely under appropriate medical supervision.) | n.a. |
| 11* | Withdrawal, as manifested by either of the following:  ● The characteristic opioid withdrawal syndrome (refer to Criteria A and B of the criteria set for opioid withdrawal).  ● Opioids (or a closely related substance) are taken to relieve or avoid withdrawal symptoms. (Note: This criterion is not considered to be met for those individuals taking opioids solely under appropriate medical supervision.) | n.a. |
| 2 - 3 criteria: mild \| 4 - 5 criteria: moderate \| ≥ 6 criteria: severe | | |
| *DO NOT apply if opioids are taken as prescribed/under medical supervision | | |

Data source: ESA – 2015, Germany.
